# Clinical characteristics of patients with Alzheimer’s disease and caregiver burden, mental health, and sleep quality: A cross-sectional comparative study in psychiatric hospitals and nursing homes

**DOI:** 10.1097/MD.0000000000049761

**Published:** 2026-07-17

**Authors:** Ke Chen, Yanfeng Deng, Dong Yan, Jing Wang, Yan Ding

**Affiliations:** a Department of Medical Affairs, Shanghai Mental Health Center Pujiang Hospital (Minhang Mental Health Center), Shanghai, China; b Department of Medical Affairs, Baoshan Mental Health Center, Shanghai, China.

**Keywords:** Alzheimer’s disease, caregiver burden, mental health, nursing Home, psychiatric hospital, sleep quality

## Abstract

As Alzheimer’s disease (AD) care shifts to institutions, differences between psychiatric hospitals and nursing homes remain unclear. We compared AD patients and professional caregivers across these settings and explored institution-specific links between caregiver burden, mental health, and sleep quality. A cross-sectional study (January to November 2024) enrolled 121 AD patients and 123 caregivers from 2 psychiatric hospitals and 2 nursing homes in Shanghai. Assessments used standardized scales. Analyses included nonparametric tests, χ^*2*^ tests, Spearman correlations, and stepwise regression. Post hoc power was adequate for moderate-to-large effects. AD patients in psychiatric hospitals are more severe, and caregivers face greater burden and psychological risk. Tailored, institution-specific mental health interventions are needed. Psychiatric hospital patients had poorer cognition and greater functional dependence (*P* < .001). Hospital caregivers reported higher burden, depression, anxiety, and worse sleep in specific domains (*P* < .05; effect sizes *r* = 0.18–0.47). Burden correlated positively with depression, anxiety, and poor sleep (*r* = 0.397–0.458). In the total sample, anxiety and poor sleep predicted burden (adjusted *R*^2^ = 0.285). In psychiatric hospitals, sleep quality was the dominant predictor (β = 0.483). In nursing homes, anxiety and workload predicted burden (adjusted *R*^2^ = 0.418); sleep quality was a negative predictor (β = −0.222), likely from floor effects and adaptive coping.

## 1. Introduction

With the accelerating global population aging, the prevalence of Alzheimer’s disease (AD) – the leading cause of dementia in the elderly – is rising rapidly, posing a severe public health challenge.^[1]^ China’s dementia population is projected to reach approximately 16.46 million by 2030.^[2]^ AD patients present progressive cognitive decline and loss of daily living abilities, often accompanied by Behavioral and Psychological Symptoms of Dementia (BPSD), which not only impair their quality of life but also exert substantial physical and mental stress on caregivers.^[3,4]^ Previous studies^[5]^ have confirmed that increased caregiver burden can damage caregivers’ mental health and reduce care quality, potentially worsening patients’ conditions. Recent years have seen psychiatric care shift from closed inpatient models toward community-institution collaboration, imposing new demands on long-term care for patients with chronic mental disorders such as AD.^[6,7]^ Meanwhile, AD patients frequently present multiple comorbidities (e.g., hypertension, diabetes, cardiovascular disease), which increase care complexity and caregiver burden.^[8,9]^ As family care capacity declines and demand for professional care rises, Alzheimer’s care has increasingly moved to specialized settings like psychiatric hospitals and nursing homes. Yet most studies treat institutional caregivers as a homogeneous group or focus on a single facility type, overlooking how different care settings may shape distinct caregiver stress mechanisms.^[10,11]^ Most studies on AD caregiver burden have mainly focused on home-based caregivers; few studies have compared caregivers in psychiatric hospitals and nursing homes, despite the fundamental differences in the nature, patient characteristics, and care models of these 2 types of institutions. This research gap results in a lack of specific caregiver support interventions, limiting their effectiveness.

This study primarily aims to compare the multidimensional profiles of AD patients and professional caregivers between psychiatric hospitals and nursing homes, and secondarily to identify factors influencing caregiver burden and their institutional heterogeneity. Based on a literature review and preliminary observations, we propose 3 hypotheses: AD patients in psychiatric hospitals have poorer cognition and greater daily living dependence, while those in nursing homes show more severe behavioral and psychological symptoms. Caregivers in psychiatric hospitals experience heavier burden, worse mental health (depression and anxiety), and poorer sleep quality. The determinants of caregiver burden differ across settings: sleep quality mainly predicts burden in psychiatric hospitals, whereas anxiety and objective workload (monthly/daily caregiving hours and number of care recipients) drive burden in nursing homes.

Using a cross-sectional design, this study compares AD patients and their caregivers across sociodemographic, clinical, cognitive, caregiving, and mental health dimensions. Spearman correlation and stepwise regression analyses are used to explore mechanisms of psychological distress, providing empirical evidence for targeted interventions.

## 2. Materials and methods

### 2.1. Study design and subjects

A cross-sectional study was conducted from January to November 2024. AD patients and their primary professional caregivers were recruited from 2 psychiatric hospitals (Minhang Mental Health Center, Baoshan Mental Health Center) and 2 nursing homes (Minhang One Family Nursing Home, Fuxing Xingbao Pujiang Senior Living Community) in Shanghai.

*Sample size estimation*: Sample size was calculated using G Power 3.1 (Heinrich-Heine-Universität Düsseldorf) assuming a moderate effect size (Cohen *d* = 0.5), α = 0.05, and power = 0.80 for the primary outcome (difference in total CBI scores between institution types), the required sample size was 128. The final sample of 123 caregivers (54 from psychiatric hospitals, 69 from nursing homes) fell slightly short of this target but remained adequate for detecting moderate-to-large effects. Post hoc power analysis indicated that the psychiatric hospital subgroup (n = 54) had 80% power for a moderate effect (*f*^2^ = 0.15), and the nursing home subgroup (n = 69) had 80% power for a small-to-moderate effect (*f*^2^ = 0.10). Thus, the subgroup regression results are acceptably robust, although their capacity to detect small effects is limited; findings should be interpreted with caution.

*Sampling strategy*: A convenience sampling method was adopted. The 2 psychiatric hospitals are district-level mental health centers in Shanghai, serving AD patients from their own and neighboring districts. The 3 nursing homes are large-scale private or public–private facilities in Minhang District that admit AD patients. Although nonrandom, the selected institutions are reasonably representative of Shanghai’s mental health and elderly care system. Nevertheless, since all participants were recruited solely from Shanghai, selection bias cannot be ruled out, and generalizability requires caution.

### 2.2. Inclusion and exclusion criteria

#### 2.2.1. Patient criteria

*Inclusion criteria*: met the diagnostic criteria for AD dementia in the World Health Organization’s International Classification of Diseases (ICD-10); aged 60–90 years; disease duration ≥ 6 months since diagnosis; and had a primary caregiver and was willing to cooperate with the study, with written informed consent provided by patients or their legal guardians.

*Exclusion criteria*: comorbid with other psychiatric disorders; unwilling to participate in the study; failed to meet the above inclusion criteria.

#### 2.2.2. Caregiver criteria

*Inclusion criteria*: served as the primary caregiver of the AD patient; aged ≥ 18 years with a caregiving duration ≥ 3 months; had a clear understanding of the patient’s condition and could provide reliable information; and voluntarily participated and provided written informed consent.

*Exclusion criteria*: had language or communication barriers that prevented completing questionnaires; unwilling to cooperate with the survey.

### 2.3. Ethical approval

This study was approved by the ethics committee of Shanghai Minhang Mental Health Center (Approval No. MH-MHC-2024-001) and conducted in accordance with the Declaration of Helsinki. All participants (or patients’ legal guardians) provided written informed consent, and the privacy rights of all subjects were strictly protected throughout the study.

### 2.4. Research instruments and variable definitions

#### 2.4.1. General information questionnaire

Self-designed by the research team, the questionnaire included 2 parts:

(1) Patient part: Age, gender, marital status, education level, alcohol use history, smoking history, number of chronic physical diseases, disease duration (months).
(2) Caregiver part: Age, gender, marital status, education level, monthly income (RMB), chronic physical disease history, number of care recipients, caregiving duration (months), caregiving days per month, daily caregiving hours (h).

#### 2.4.2. Patient assessment scales

(1) Mini-Mental State Examination (MMSE): Assessed cognitive function (30 items, 0–30 points), with higher scores indicating better cognitive function. Cognitive impairment was defined as a score < 17 for illiterate patients, < 20 for primary school educated patients, and < 24 for patients with junior high school education and above.^[12]^
(2) Activities of Daily Living Scale (ADL): Assessed daily living ability (14 items, 14–56 points), with higher scores indicating worse ability and higher dependency.^[13]^
(3) Behavioral Pathology in Alzheimer’s Disease Rating Scale (BEHAVE-AD): Assessed BPSD (25 items, 0–100 points), with higher scores indicating more severe behavioral and psychological symptoms.^[14]^

#### 2.4.3. Caregiver assessment scales

(1) Caregiver Burden Inventory (CBI): Evaluated caregiver burden (24 items, 0–96 points), including 5 dimensions: time-dependence, developmental, physical, social, and emotional burden. Higher scores indicated greater burden (0–32: mild; 33–64: moderate; 65–96: severe).^[15]^
(2) Patient Health Questionnaire-9 (PHQ-9): Assessed depressive symptoms (9 items, 0–27 points). Scores ≥ 5 indicated depressive symptoms (5–9: mild; 10–14: moderate; ≥15: moderate-severe to severe).^[16]^
(3) Generalized Anxiety Disorder-7 (GAD-7): Assessed anxiety symptoms (7 items, 0–21 points). Scores ≥ 5 indicated anxiety symptoms (5–9: mild; 10–14: moderate; ≥15: severe).^[17]^
(4) Pittsburgh Sleep Quality Index (PSQI): Assessed sleep quality in the past month (7 components, 0–21 points). Scores ≥ 7 indicated poor sleep quality; higher scores indicated worse sleep quality.^[18]^

### 2.5. Data collection and statistical analysis

#### 2.5.1. Data collection

Trained psychiatrists and nurses conducted face-to-face interviews in quiet institutional settings (meeting rooms, consultation rooms). For patients, the MMSE, ADL, and BEHAVE-AD were completed. If patients could not cooperate, relevant information was supplemented by caregivers and medical records. For caregivers, the general information questionnaire and 4 scales (CBI, PHQ-9, GAD-7, PSQI) were completed under guidance, with each questionnaire taking 20–30 minutes to finish. All questionnaires were checked on-site for completeness, with missing items supplemented immediately to ensure a 100% valid response rate.

#### 2.5.2. Statistical analysis

SPSS 26.0 software (IBM Corporation) was used for statistical analysis. The Shapiro–Wilk test was applied to test the normality of continuous variables. Continuous variables were expressed as median (interquartile range) [*M (P25, P75*)] and compared using the Mann–Whitney *U* test. Categorical variables were expressed as frequency (percentage) [n (%)] and compared using the χ^2^ test. Correlations between caregiver burden, anxiety, depression, and sleep quality were analyzed using Spearman’s rank correlation analysis. Stepwise multiple linear regression analysis was used to explore the influencing factors of caregiver burden. A 2-sided *P* < .05 was considered statistically significant.

## 3. Results

### 3.1. Baseline characteristics of AD patients

A total of 121 AD patients were included in the study (66 from nursing homes, 55 from psychiatric hospitals).

Significant differences were found in demographic and disease-related indicators, and clinical scale scores between the 2 groups. The nursing home group had a higher median age, a higher proportion of widowed patients and patients with primary school education, and a lower proportion of illiterate patients (all *P* < .05). The psychiatric hospital group had more chronic physical diseases (*P* < .001). The nursing home group had higher MMSE and BEHAVE-AD scores (indicating better cognitive function and more severe BPSD), while the psychiatric hospital group had a higher ADL score (indicating higher daily living dependency; all *P* < .001). No significant differences were found in gender, alcohol use, smoking history, or disease duration between the 2 groups (all *P* > .05; Table 1).

**Table 1:** Baseline characteristics of AD patients [*M (P25, P75*) or n (%)].

Item	Psychiatric hospital (n = 55)	Nursing home (n = 66)	χ^*2*^*/Z*	*P*
Gender			0.498	.48
Male	20 (36.36%)	20 (30.30%)		
Female	35 (63.64%)	46 (69.70%)		
Age	79.0 (75.0–83.0)	87.5 (80.8–91.3)	−4.866	<.001
Marital status			16.636	.001
Married	28 (50.91%)	11 (16.67%)		
Widowed	23 (41.82%)	48 (72.73%)		
Divorced	2 (3.64%)	5 (7.58%)		
Unmarried	2 (3.64%)	2 (3.03%)		
Education level			25.465	<.001
Illiterate	11 (20.00%)	1 (1.52%)		
Primary school	16 (29.09%)	34 (51.52%)		
Junior high school	19 (34.55%)	11 (16.67%)		
High school/vocational	8 (14.55%)	8 (12.12%)		
College or above	1 (1.82%)	12 (18.18%)		
Alcohol use			2.51	.113
No	51 (92.73%)	65 (98.48%)		
Yes	4 (7.27%)	1 (1.52%)		
Smoking history			0.374	.541
No	53 (96.36%)	62 (93.94%)		
Yes	2 (3.64%)	4 (6.06%)		
Number of chronic physical diseases	2.0 (1.0–2.0)	3.0 (2.0–4.0)	−5.245	<.001
Disease duration (mo)	64.0 (28.0–114.0)	39.0 (23.0–78.5)	−1.848	.065
MMSE total score	0.0 (0.0–7.0)	16.0 (7.0–23.0)	−6.352	<.001
BEHAVE-AD total score	0.0 (0.0–2.0)	5.0 (1.0–11.0)	−5.210	<.001
ADL total score	56.0 (48.0–56.0)	38.0 (31.0–43.0)	−7.177	<.001

AD = Alzheimer’s disease, ADL = Activities of Daily Living Scale, BEHAVE-AD = Behavioral Pathology in Alzheimer’s Disease Rating Scale, MMSE = Mini-Mental State Examination.

### 3.2. Baseline characteristics of caregivers

A total of 123 caregivers were included in the study (69 from nursing homes, 54 from psychiatric hospitals).

Significant differences were found in demographic, caregiving, and health characteristics between the 2 caregiver groups. The nursing home group had a higher proportion of female caregivers and caregivers with junior high school education, with no illiterate caregivers (all *P *< .05). Caregivers in psychiatric hospitals had more care recipients, longer daily caregiving hours, higher monthly income, and a certain proportion of chronic physical diseases (all *P* < .05). No significant differences were found in age, marital status, or caregiving duration between the 2 groups (all *P* > .05; Table 2).

**Table 2:** Baseline characteristics of AD caregivers [*M (P25, P75*) or n (%)].

Item	Psychiatric hospital (n = 54)	Nursing home (n = 69)	χ^*2*^*/Z*	*P*
Gender			11.206	.001
Male	15 (27.78%)	4 (5.80%)		
Female	39 (72.22%)	65 (94.20%)		
Age	56.0 (52.8–59.0)	55.0 (51.0–57.0)	−1.477	.140
Marital status			2.993	.224
Married	52 (96.30%)	65 (94.20%)		
Widowed	0 (0.00%)	3 (4.35%)		
Divorced	2 (3.70%)	1 (1.45%)		
Unmarried	0 (0.00%)	0 (0.00%)		
Education level			12.906	.012
Illiterate	6 (11.11%)	0 (0.00%)		
Primary school	23 (42.59%)	21 (30.43%)		
Junior high school	20 (37.04%)	39 (56.52%)		
High school/vocational	5 (9.26%)	7 (10.14%)		
College or above	0 (0.00%)	2 (2.90%)		
Number of care recipients	5.0 (4.0–6.0)	3.0 (3.0–3.0)	−7.154	<.001
Caregiving duration (mo)	12.0 (7.8–30.0)	19.0 (12.0–64.6)	−1.884	.060
Caregiving d/mo	30.0 (30.0–30.0)	30.0 (30.0–30.0)	−2.995	.003
Daily caregiving hours	24.0 (24.0–24.0)	12.0 (12.0–12.0)	−9.253	<.001
Monthly income (RMB)	8000.0 (7800.0–8550.0)	5500.0 (5500.0–5500.0)	−9.319	<.001
Chronic physical disease			3.929	.047
No	51 (94.44%)	69 (100.00%)		
Yes	3 (5.56%)	0 (0.00%)		

AD = Alzheimer’s disease.

### 3.3. Comparison of caregiver burden, mood, and sleep quality scores

Caregivers in psychiatric hospitals had significantly higher CBI total scores and emotional burden dimension scores than those in nursing homes (*P* < .05), with no significant differences in other CBI dimensions (all *P *> .05). The total CBI score (*r* = 0.18) and most other dimensions had small effect sizes, suggesting that statistically significant between-group differences on some indicators were accompanied by small effects. PHQ-9 and GAD-7 scores were significantly higher in the psychiatric hospital group (all *P* < .001). Both PHQ-9 (*r* = 0.42) and GAD-7 (*r* = 0.47) demonstrated moderate effect sizes, suggesting clinically meaningful differences in depression and anxiety between the 2 caregiver groups. There was no significant difference in PSQI total scores between the 2 groups, but the psychiatric hospital group had higher scores in subjective sleep quality, sleep latency, sleep duration, and daytime dysfunction (all *P* < .05). Daytime dysfunction (*r* = 0.33) had a moderate effect, whereas sleep latency (*r* = 0.24), emotional burden (*r* = 0.21), and sleep duration (*r* = 0.20) had small-to-moderate effects (Table 3).

**Table 3:** Comparison of caregiver burden, mood, and sleep quality scores [*M (P25, P75*)].

Item	Psychiatric hospital (n = 54)	Nursing home (n = 69)	*Z*	*P*	*r*
CBI total score	39.0 (26.5–54.3)	33.0 (30.0–36.5)	−2.020	.043	0.18
Time dependence burden	7.4 (4.0–13.0)	5.0 (5.0–8.0)	−1.610	.107	0.15
Developmental burden	5.0 (2.0–7.0)	5.0 (4.0–5.0)	−1.142	.253	0.10
Physical burden	4.0 (2.0–6.0)	4.0 (2.0–4.0)	−1.267	.205	0.11
Social burden	13.0 (12.0–16.0)	12.0 (12.0–14.0)	−1.269	.204	0.11
Emotional burden	11.0 (3.8–15.3)	6.0 (6.0–9.5)	−2.282	.022	0.21
PHQ-9 total score	3.0 (0.0–7.0)	0.0 (0.0–2.0)	−4.702	<.001	0.42
GAD-7 total score	1.0 (0.0–4.0)	0.0 (0.0–0.0)	−5.195	<.001	0.47
PSQI total score	5.5 (3.0–12.0)	5.0 (4.0–6.0)	−1.436	.151	0.13
Subjective sleep quality	1.0 (0.0–2.0)	1.0 (0.0–1.0)	−2.010	.044	0.18
Sleep latency	2.0 (1.0–2.3)	1 (1.0–2.0)	−2.635	.008	0.24
Sleep duration	1.0 (1.0–2.0)	1.0 (1.0–2.0)	−2.184	.029	0.20
Habitual sleep efficiency	0.0 (0.0–3)	1.0 (0.0–1.5)	−0.386	.699	0.03
Sleep disturbances	1.0 (1.0–1.0)	1.0 (1.0–1.0)	−1.654	.098	0.15
Use of sleeping medication	0.0 (0.0–0.0)	0.0 (0.0–0.0)	−1.256	.209	0.11
Daytime dysfunction	1.0 (0.0–2.0)	0.0 (0.0–0.5)	−3.677	<.001	0.33

*r* values of 0.10 to 0.29 indicate a small effect, 0.30 to 0.49 a medium effect, and ≥0.50 a large effect.

CBI = Caregiver Burden Inventory, GAD-7 = Generalized Anxiety Disorder-7, PHQ-9 = Patient Health Questionnaire-9, PSQI = Pittsburgh Sleep Quality Index.

### 3.4. Comparison of caregiver mental health clinical severity grades

All severe caregiver burden cases (3 cases) were concentrated in the psychiatric hospital group, with no significant difference in the distribution of mild/moderate/severe burden (*P* > .05). The proportion of caregivers with depressive symptoms (PHQ-9 ≥ 5) was 37.04% in the psychiatric hospital group and 4.35% in the nursing home group (*P* < .001). The proportion of caregivers with anxiety symptoms (GAD-7 ≥ 5) was 22.22% in the psychiatric hospital group and 1.45% in the nursing home group (*P* < .001). The proportion of caregivers with poor sleep quality (PSQI ≥ 7) was 46.30% in the psychiatric hospital group and 24.64% in the nursing home group (*P* < .05; Table 4).

**Table 4:** Comparison of caregiver mental health clinical severity grades [n (%)].

Assessment tool & clinical grade	Psychiatric hospital (n = 54)	Nursing home (n = 69)	χ^*2*^	*P*	Cramér’s *V*
CBI			3.952	.139	0.18
Mild	20 (37.04%)	28 (40.60%)			
Moderate	31 (57.41%)	41 (59.40%)			
Severe	3 (5.55%)	0 (0.0%)			
PHQ-9			21.734	<.001	0.42
None	34 (62.96%)	66 (95.65%)			
Mild	15 (27.78%)	3 (4.35%)			
Moderate	4 (7.41%)	0 (0.00%)			
Moderate–severe	0 (0.00%)	0 (0.00%)			
Severe	1 (1.85%)	0 (0.00%)			
GAD-7			17.578	.001	0.38
None	42 (77.78%)	68 (98.55%)			
Mild	11 (20.37%)	0 (0.00%)			
Moderate	1 (1.85%)	0 (0.00%)			
Severe	0 (0.00%)	1 (1.45%)			
PSQI			6.319	.012	0.23
Good	29 (53.70%)	52 (75.36%)			
Poor	25 (46.30%)	17 (24.64%)			

CBI = Caregiver Burden Inventory, GAD-7 = Generalized Anxiety Disorder-7, PHQ-9 = Patient Health Questionnaire-9, PSQI = Pittsburgh Sleep Quality Index.

### 3.5. Correlation between caregiver burden and anxiety, depression, sleep quality

Spearman correlation analysis showed that the CBI total score was significantly positively correlated with GAD-7 (*r* = 0.397), PHQ-9 (*r* = 0.403), and PSQI (*r* = 0.458; all *P* < .001). Emotional burden had the strongest correlation with anxiety (*r* = 0.515) and depression (*r* = 0.564), while time-dependence burden had the highest correlation with sleep quality (*r* = 0.491). Social burden had no significant correlation with anxiety, depression, or sleep quality (all *P* > .05; Table 5).

**Table 5:** Spearman correlation between CBI total score/dimensions and anxiety, depression, sleep (N = 123).

Variable	GAD-7 total	PHQ-9 total	PSQI total
CBI total	0.397**	0.403**	0.458**
Time dependence burden	0.357**	0.485**	0.491**
Developmental burden	0.326**	0.264**	0.282**
Physical burden	0.233**	0.205*	0.186*
Social burden	−0.113	−0.063	−0.093
Emotional burden	0.515**	0.564**	0.459**

CBI = Caregiver Burden Inventory, GAD-7 = Generalized Anxiety Disorder-7, PHQ-9 = Patient Health Questionnaire-9, PSQI = Pittsburgh Sleep Quality Index.

* *P* < .05.

** *P* < .001.

### 3.6. Influencing factors of caregiver burden (stepwise regression)

#### 3.6.1. Total sample

With the CBI total score of all caregivers as the dependent variable and variables with significant differences in univariate analysis as independent variables, stepwise regression was performed. The results showed that anxiety (GAD-7 total score) and poor sleep quality (PSQI total score) entered the final model and were both significant positive predictors (both *P* < .01; Table 6).

**Table 6:** Stepwise regression for total sample CBI influencing factors (n = 123).

Independent variable	*B*	SE	β	*t*	*P*	95% CI
Constant	28.620	2.119		13.505	<.001	[24.47, 32.77]
GAD-7 total	1.552	0.493	0.312	3.149	.002	[0.59, 2.52]
PSQI total	1.049	0.359	0.290	2.921	.004	[0.35, 1.75]

*R*^2^ = 0.297, Adjusted *R*^2^ = 0.285; *F* = 25.336, *P* < .001.

CBI = Caregiver Burden Inventory, CI = confidence interval, GAD-7 = Generalized Anxiety Disorder-7, PSQI = Pittsburgh Sleep Quality Index, SE = standard error.

#### 3.6.2. Subgroup analysis

Grouped by institution type, separate stepwise regressions were performed with the CBI total score of each group as the dependent variable. The regression models showed significant heterogeneity between the 2 groups (Table 7).

**Table 7:** Stepwise regression for caregiver burden in different institution subgroups.

Group	Independent variable	*B*	SE	β	*t*	*P*	95% CI
Psychiatric hospital (n = 54)	Constant	26.594	3.573		7.443	<.001	[19.42, 33.77]
	PSQI total	1.764	0.423	0.483	4.166	<.001	[0.91, 2.62]
	Education (high school/vocational school vs other)	15.252	6.964	0.254	2.190	.033	[1.26, 29.24]
Nursing home(n = 69)	Constant	−2.044	10.049		−0.203	.839	[−22.13, 18.04]
	PSQI total	−0.813	0.404	−0.222	−2.016	.048	[−1.62, −0.01]
	GAD-7 total	2.588	0.426	0.682	6.080	<.001	[1.73, 3.45]
	Caregiving d/mo	1.257	0.260	0.579	4.844	<.001	[0.73, 1.78]
	Gender (male vs female)	−6.429	3.133	−0.198	−2.052	.044	[−12.70, −0.16]
	Daily caregiving hours	0.784	0.193	0.443	4.069	<.001	[0.40, 1.17]
	Number of care recipients	1.541	0.530	0.352	2.907	.005	[0.48, 2.60]

CI = confidence interval, GAD-7 = Generalized Anxiety Disorder-7, PSQI = Pittsburgh Sleep Quality Index, SE = standard error.

(1) Psychiatric hospital group (n = 54): The final model included sleep quality (PSQI total score) and education level (high school/vocational school vs other). Sleep quality was the main positive predictor (β = 0.483, *P* < .001). Caregivers with a high school or vocational school education had a significantly heavier burden (β = 0.254, *P* = .033). The adjusted *R*^2^ was 0.336, indicating that sleep quality and education level together explained 33.6% of the variance in caregiver burden.
(2) Nursing home group (n = 69): The final model included 6 variables and showed a relatively strong explanatory power (adjusted *R*^2^ = 0.418). Anxiety (GAD-7 total score) was the strongest predictor (β = 0.682, *P* < .001). Objective caregiving workload (monthly caregiving days, daily caregiving hours, and number of care recipients) significantly increased burden. Gender was a negative predictor (β = −0.198, *P* = .044), suggesting that male caregivers experienced lower burden. Sleep quality (PSQI total score) was a significant negative predictor (β = −0.222, *P* = .048) – that is, poorer sleep quality was associated with lower burden—a finding that requires cautious interpretation.

#### 3.6.3. Homogeneity of variance test and floor effect

To examine whether the negative prediction of sleep quality for caregiver burden in the nursing home group was related to data distribution characteristics, the Brown–Forsythe test (median-based test for homogeneity of variance) was used to compare the variance of CBI scores between the 2 groups. The results showed that the mean CBI score was 40.91 (SD = 17.58) in the psychiatric hospital group and 33.96 (SD = 7.66) in the nursing home group. The Brown–Forsythe statistic was 49.488 (*P* < .001), indicating unequal variances between the 2 groups. The standard deviation of CBI scores in the nursing home group was only 43.6% of that in the psychiatric hospital group, reflecting a significantly narrower range of variation and suggesting a “floor effect” in caregiver burden among the nursing home group (Table 8).

**Table 8:** Descriptive statistics and homogeneity of variance test for total CBI scores between the 2 caregiver groups.

Group	N	Mean	SD	Min	Max
Psychiatric hospital	54	40.91	17.58	0	88
Nursing home	69	33.96	7.66	12	57
Brown–Forsythe test	Levene statistic = 49.488	df1 = 1, df2 = 121	*P* < .001		

CBI = Caregiver Burden Inventory, SD = standard deviation.

### 3.7. Exploratory analysis of factors influencing sleep quality

To better understand sleep problems in caregivers, stepwise regression was performed with the PSQI total score as the dependent variable. The final model showed that anxiety, depression, caregiving days per month, gender, caregiver burden, and education level (illiterate vs others) entered the final model, jointly explaining 58.8% of the variance, and all were significant predictors (all *P* < .05; Table 9).

**Table 9:** Stepwise regression for sleep quality (PSQI total score) influencing factors of AD caregivers (N = 123).

Independent variable	*B*	SE	β	*t*	*P*	95% CI
Constant	−8.890	2.541		−3.499	.001	[−13.91, −3.91]
GAD-7 total	0.550	0.115	0.401	4.768	<.001	[0.33, 0.78]
Caregiving days/month	0.298	0.081	0.225	3.659	<.001	[0.14, 0.46]
PHQ-9 total	0.327	0.074	0.329	4.435	<.001	[0.18, 0.47]
Gender (female vs male)	1.763	0.607	0.173	2.905	.004	[0.57, 2.95]
CBI total	0.046	0.020	0.166	2.331	.021	[0.01, 0.09]
Education (illiterate vs others)	−2.049	1.025	−0.120	−1.999	.048	[−4.06, −0.04]

AD = Alzheimer’s disease, CBI = Caregiver Burden Inventory, CI = confidence interval, GAD-7 = Generalized Anxiety Disorder-7, PHQ-9 = Patient Health Questionnaire-9, PSQI = Pittsburgh Sleep Quality Index, SE = standard error.

## 4. Discussion

AD patients often present with cognitive decline, self-care deficits, and multiple chronic comorbidities, requiring long-term care that exerts substantial burden on caregivers and indirectly affects patients’ quality of life.^[19]^ This systematic investigation and comparative analysis revealed that professional caregivers in both psychiatric hospitals and nursing homes experience a certain degree of caregiving burden, accompanied by anxiety, depression, and poor sleep quality. Significant differences existed between the 2 settings in care context, patient characteristics, and stress mechanisms, and key influencing factors of caregiver burden were identified, providing empirical evidence for establishing a targeted caregiver support system.

CBI results showed median total burden scores of 39.0 (26.5–54.3) in psychiatric hospital caregivers and 33.0 (30.0–36.5) in nursing home caregivers. Both scores are higher than the Chinese norm (24.2 ± 5.8),^[20]^ but lower than the results reported by Chen et al (59.16 ± 13.65),^[21]^ and close to the findings of Yang (40.56 ± 13.16).^[22]^ Overall, the burden level of AD caregivers in this study is moderate. Burden levels ranked from high to low as social burden, emotional burden, time-dependent burden, developmental burden, and physical burden, which differs from the results of Liu et al,^[23]^ whose study focused on family caregivers. This difference may be due to the fact that all participants in this study are professional nursing staff. Most caregivers were female (74.22% in hospitals, 94.20% in nursing homes), which is consistent with previous research and may be related to traits commonly associated with women such as greater patience and attentiveness.^[24]^ Caregivers were mainly over 50 years old, while most patients were over 70 years old, reflecting the intergenerational care of older adults by older caregivers and the pressure on Shanghai’s elderly care and medical systems. Existing challenges in institutional AD care include uneven institutional development, insufficient professional competence of caregivers, and limited dementia-specific training.^[25]^ Evidence suggests^[26]^ that the professional skill level and coping strategy proficiency of caregivers are negatively correlated with their perceived burden. Therefore, strengthening specialized care training and coping guidance for caregivers is critical to reducing their burden and improving care quality.

Systematic differences in patient characteristics between the 2 institutions reveal an implicit triaging mechanism in China’s long-term care system, which is based on disease severity and problem type – a phenomenon aligned with the globally advocated tiered care model.^[27,28]^ Psychiatric hospitals admitted relatively younger patients with more severe cognitive impairment, higher daily living dependency, and more physical comorbidities, focusing on acute treatment and severe symptom management. Nursing homes cared for older patients with better cognitive function but more persistent behavioral and psychological symptoms, which is suitable for stable patients who are difficult to manage at home. It is worth noting that negative psychological states in elderly patients with chronic diseases may worsen BPSD. Dörtkardeş et al^[29]^ found that perceived social exclusion was significantly positively associated with hopelessness in this population. For AD residents in nursing homes, cognitive decline can reduce social interaction and increase feelings of exclusion, thereby triggering depression and anxiety, which indirectly heighten caregiver burden. Thus, psychological support for AD patients – such as reducing perceived social exclusion – may benefit both patients and caregivers. Such differentiation has shaped the stress sources and coping patterns of caregivers in the 2 settings, supporting the development of targeted intervention measures for caregivers.

This study found that caregivers in psychiatric hospitals bear higher psychological risks and caregiving burden, likely due to the different work models of the 2 institutions. Nursing home caregivers work in shifts and have regular time off, enabling adequate rest and recovery. In contrast, psychiatric hospital caregivers – despite higher pay – face heavier workloads, including more care recipients and longer daily caregiving hours (mostly 24-hour shifts). Accordingly, they scored significantly higher on total burden, emotional burden, depression, and anxiety. Clinical assessment revealed that 37.04% and 22.22% of hospital caregivers had mild or greater depression and anxiety symptoms, respectively, and all severe burden cases were in this group, highlighting their psychological vulnerability. Thus, economic compensation only partly mitigates caregiver stress and cannot fully offset the emotional exhaustion caused by high-intensity care. From a data-driven perspective, psychological distress in psychiatric hospital caregivers can be attributed to the cumulative effect of high-intensity caregiving workload. In this study, their median daily caregiving time was 24 hours, and median care recipients numbered 5 – far exceeding the nursing home group’s 12 hours and 3 persons (Table 2). This high-intensity pattern directly led to sleep deprivation (46.30% reporting poor sleep quality), and emotional burden was strongly correlated with depression and anxiety (*R* = 0.564 and 0.515, respectively; Table 5). Regression analysis further confirmed sleep quality as a strong predictor of caregiver burden in this group (β = 0.483; Table 7). Sleep quality analysis further showed that hospital caregivers had poorer subjective sleep quality, longer sleep latency, and more severe daytime dysfunction, confirming the presence of chronic stress in this group. Sleep deprivation, in turn, exacerbates mood symptoms and perceived burden, creating a vicious cycle.^[30]^

Correlation and regression analyses identified the key pathways in the formation of caregiver burden, providing a basis for precision interventions. Emotional burden showed the strongest correlation with anxiety (*r* = 0.515) and depression (*r* = 0.564), indicating that intrinsic emotional distress – rather than physical or time demands – is the core driver of emotional problems in caregivers. This finding aligns with previous research^[31]^ and suggests shifting the focus of caregiver interventions from reducing physical load to alleviating emotional stress, particularly in psychiatric hospital settings where disease progression is irreversible. Regression analysis for the total sample showed that anxiety and poor sleep quality were significant positive predictors of caregiver burden. Subgroup analyses offered greater practical insights for targeted interventions. For psychiatric hospital caregivers, burden was primarily influenced by sleep quality – likely due to the 24-hour care model – thus interventions should prioritize improving sleep quality. Education level also affected caregiver burden, which is consistent with previous studies.^[32-34]^ In nursing homes, caregiver burden was influenced by multiple factors, with anxiety exerting the strongest effect (β = 0.682), which probably stems from the continuous pressure of managing patients’ persistent behavioral and psychological symptoms. Hence, interventions for nursing home caregivers should balance emotional support and training in behavioral and psychological symptom management skills. Gender remained a significant influencing factor, with female caregivers reporting higher burden, which is supported by existing conclusions.^[35]^ This may be because women are generally more emotionally sensitive and have richer and more delicate emotional perceptions, making them more susceptible to psychological burden.^[35-38]^ Longer monthly caregiving days and daily caregiving hours were associated with higher burden levels, consistent with existing research,^[37,39]^ indicating that longer direct caregiving time leads to greater caregiver burden.^[40]^ For dementia patients with declining self-care abilities, caregivers need to invest more time and effort in daily care.^[41]^ Studies indicate that caregiving exceeding 16 hours daily disrupts sleep,^[42]^ and caregiving time of <4 hours per day significantly reduces caregiver burden.^[43]^ Long-term high-intensity caregiving compromises caregivers’ physical and psychological health, manifesting as anxiety, depression, and sleep disturbances.^[44]^ Reduced personal time limits caregivers’ social activities and rest; unmet rest and social needs, coupled with excessive caregiving burden, further impair caregivers’ physical and mental health and care capacity, creating a vicious cycle. To care for AD patients, caregivers often suspend their social activities, maintain constant vigilance, and experience high levels of mental tension. Combined with a lack of social support, they are prone to negative emotions such as depression, anxiety, and loneliness,^[45]^ and without effective avenues for emotional release, their caregiving burden continues to increase. Notably, sleep quality was a negative predictor of caregiver burden in the nursing home group (β = −0.222, *P* = .048), meaning poorer sleep was associated with lower self-reported burden. This paradoxical finding may have 2 explanations. First, adaptive professional coping strategies: nursing home caregivers work fixed shifts (12 h/d) with team support, which may foster adaptive approaches such as fragmented sleep (e.g., short rest breaks) or cognitive reappraisal, partially buffering the impact of sleep loss on perceived burden. Second, floor effect: the median CBI score in this group was low (33 points) with narrow variability, potentially limiting statistical power. The Brown–Forsythe test showed that CBI variance in the nursing home group was significantly smaller than that in the psychiatric hospital group (Levene statistic = 49.488, *P* < .001), and its standard deviation (7.66) was only 43.6% of that of the psychiatric hospital group (17.58), indicating restricted variability. Insufficient variance in the dependent variable reduces statistical power, making it difficult to reliably detect true associations and potentially causing unstable or reversed regression coefficients. Moreover, the primary drivers of burden in the nursing home group were anxiety (GAD-7 β = 0.682) and objective workload (β = 0.352–0.579); these strong predictors may have masked the weak effect of sleep quality. Therefore, this result should not be overinterpreted as “poor sleep reduces burden” but rather as a consequence of limited statistical power and the interaction of multiple predictors. This phenomenon warrants further qualitative research using larger samples to validate the findings and explore adaptive coping mechanisms among nursing home caregivers.

To build a localized precision care system for AD institutional caregivers, several recommendations are proposed based on the study results. First, establish institution-specific caregiver support systems: for psychiatric hospitals, strengthen regular mental health screening for caregivers, implement evidence-based psychological interventions, optimize shift schedules to reduce 24-hour continuous care, and provide sleep hygiene guidance. For nursing home caregivers, communication skills training could be an effective intervention entry point. Kaplan et al showed that a psychoeducational program significantly improved caregivers’ communication skills and self-esteem.^[46]^ Given that nursing home caregivers in this study need to manage BPSD in AD patients – and effective communication is a core competency for BPSD management – future interventions could draw on such psychoeducational models, adapt them to the Chinese cultural context, and thereby enhance caregivers’ ability to address patients’ behavioral problems. Meanwhile, rational manpower allocation, including increasing staffing levels and optimizing care workflows, can reduce objective caregiving workload. Second, improve sustainable professional support policies for institutional AD caregivers, including reasonable salary increases, clear career development paths, and comprehensive welfare guarantees, to enhance their professional sense of belonging and reduce turnover. Third, promote a tiered collaborative care model among psychiatric hospitals, community health service centers, and nursing homes, with psychiatric hospitals focusing on the acute treatment and complex symptom management of severe AD patients, community health service centers on the rehabilitation and regular follow-up of mild-to-moderate AD patients, and nursing homes on the long-term daily care of stable AD patients. This model can improve the efficiency of medical and elderly care resource allocation and reduce the overall care pressure on institutional caregivers.

This study has several limitations. First, the cross-sectional design precludes causal inference. Second, the sample was recruited from only 4 institutions in a specific area of Shanghai using convenience sampling, posing a high risk of selection bias. Moreover, inherent differences in disease severity between patient groups across the 2 institution types may have confounded comparisons of caregiver burden, and no multivariate adjustment was performed – a key methodological limitation. Third, the study relied mainly on self-report scales, which may introduce reporting bias. Fourth, the subgroup analyses were based on relatively small sample sizes (n = 54 and n = 69), providing insufficient statistical power to detect small effect sizes; thus, the stability of the regression models requires validation in larger samples. Future research can proceed in 3 directions. First, longitudinal follow-up studies tracking caregivers for 6 to 12 months could explore dynamic trajectories and critical transition points in burden, emotional disorders, and sleep disturbances. Second, intervention studies should test efficacy and feasibility: drawing on Kaplan et al,^[46]^ designs should be tailored to the institutional heterogeneity identified here – prioritizing sleep improvement for psychiatric hospital caregivers and anxiety management with communication skills training for nursing home caregivers. Third, future studies could integrate objective physiological measures (e.g., cortisol levels, polysomnography) with qualitative methods (e.g., in-depth interviews) to gain a multidimensional understanding of the mechanisms underlying caregivers’ psychological distress. Furthermore, including family caregivers in comparative analyses would help provide a more comprehensive picture of the AD caregiving landscape in China.

## 5. Conclusion

Significant differences exist between AD patients and their professional caregivers in psychiatric hospitals and nursing homes. Psychiatric hospital patients have more severe disease conditions and more complex care needs, and their caregivers bear heavier workloads and higher levels of anxiety, depression, and sleep disturbances. Emotional burden and anxiety are core determinants of caregiving burden, with distinct influencing mechanisms in the 2 types of institutions. Developing differentiated, mental-health-centered support systems for institutional caregivers is essential to improve the quality of AD institutional care and achieve sustainable responses to the dementia challenge in an aging society.

## Acknowledgments

The authors thank all the AD patients, professional caregivers, and staff from the participating institutions for their valuable participation and cooperation in this study.

## Author contributions

**Conceptualization:** Yan Ding.

**Data curation:** Ke Chen.

**Formal analysis:** Ke Chen, Yanfeng Deng, Yan Ding.

**Funding acquisition:** Yan Ding.

**Investigation:** Ke Chen, Yanfeng Deng, Dong Yan, Jing Wang.

**Methodology:** Ke Chen, Yanfeng Deng, Dong Yan, Yan Ding.

**Project administration:** Yan Ding.

**Resources:** Dong Yan, Jing Wang, Yan Ding.

**Software:** Ke Chen.

**Supervision:** Ke Chen, Yanfeng Deng, Dong Yan, Jing Wang, Yan Ding.

**Validation:** Ke Chen, Yanfeng Deng, Dong Yan, Jing Wang, Yan Ding.

**Visualization:** Ke Chen.

**Writing – original draft:** Ke Chen.

**Writing – review & editing:** Yanfeng Deng, Dong Yan, Jing Wang, Yan Ding.
